# Microbial production of 1-octanol: A naturally excreted biofuel with diesel-like properties

**DOI:** 10.1016/j.meteno.2014.11.001

**Published:** 2014-11-13

**Authors:** M. Kalim Akhtar, Hariharan Dandapani, Kati Thiel, Patrik R. Jones

**Affiliations:** a Department of Biochemistry, University of Turku, Tykistökatu 6B 4krs, 20520 Turku, Finland; b Department of Biochemical Engineering, University College London, Torrington Place, London WC1E 7JE, UK; c Department of Life Sciences, Imperial College London, Sir Alexander Fleming Building, London SW7 2AZ, UK

**Keywords:** 1-Octanol, Fatty alcohol, Diesel, Biofuel, Excretion

## Abstract

The development of sustainable, bio-based technologies to convert solar energy and carbon dioxide into fuels is a grand challenge. A core part of this challenge is to produce a fuel that is compatible with the existing transportation infrastructure. This task is further compounded by the commercial desire to separate the fuel from the biotechnological host. Based on its fuel characteristics, 1-octanol was identified as an attractive metabolic target with diesel-like properties. We therefore engineered a synthetic pathway specifically for the biosynthesis of 1-octanol in *Escherichia coli* BL21(DE3) by over-expression of three enzymes (thioesterase, carboxylic acid reductase and aldehyde reductase) and one maturation factor (phosphopantetheinyl transferase). Induction of this pathway in a shake flask resulted in 4.4 mg 1-octanol L^−1^ h^−1^ which exceeded the productivity of previously engineered strains. Furthermore, the majority (73%) of the fatty alcohol was localised within the media without the addition of detergent or solvent overlay. The deletion of *acrA* reduced the production and excretion of 1-octanol by 3-fold relative to the wild-type, suggesting that the AcrAB–TolC complex may be responsible for the majority of product efflux. This study presents 1-octanol as a potential fuel target that can be synthesised and naturally accumulated within the media using engineered microbes.

## Highlights

• 1-Octanol has fuel characteristics similar to that of diesel.
• A synthetic 1-octanol pathway was engineered using an octanoic acid-generating fatty acyl-ACP thioesterase.
• Deletion of *acrA* reduced the synthesis and efflux of 1-octanol.

## 1 Introduction

The demand for diesel fuel continues to increase (Cames and Helmers, 2013). In response to these demands, and amid concerns over the environmental impact of fossil fuels, intensive research efforts have been made in developing renewable and sustainable methods for the production of diesel substitutes (Peralta-Yahya et al., 2011, Steen et al., 2010). To date, biodiesel is the most extensively researched diesel fuel replacement (Atabani et al., 2012). Its synthesis traditionally involves chemical or mechanical extraction of oils from plant or algal sources followed by trans-esterification to yield either fatty acid methyl esters or ethyl esters. Innovations in microbial engineering have led to bioprocesses which obviate the need for a separate esterification step and potentially allow multiple industrial waste streams to be utilised (Kalscheuer et al., 2006, Steen et al., 2010). The major downside to biodiesel or even precursor fuels such as the recently reported bisabolane (Peralta-Yahya et al., 2011) is that they require energy intensive extraction procedures (*e.g.* usage of organic solvents for end-product isolation, high centrifugal forces for biomass recovery, physical methods for disruption of biomass) and/or further chemical modifications, and this can present a major economic barrier for the purpose of fuel commercialisation (Chisti, 2013).

Alcohols and alkanes are both highly attractive biofuel candidates as they do not require further chemical modification. In diesel engines, one particularly notorious issue with the use of alkanes is the formation of particulate matter. These carbonaceous particles, also known as soot, are attributed to incomplete combustion and have been shown in several studies to exacerbate respiratory illnesses and contribute to global warming (Bond et al., 2013, D’Amato et al., 2013). In this regard, alcohols have generated a considerable amount of interest since their increased oxygenated content can significantly stimulate the completion of combustion and thereby lower the production of particulate matter.

Herein, we evaluate the fuel and physicochemical characteristics of saturated fatty alcohols and conclude that the C8 fatty alcohol, 1-octanol, is a highly attractive biofuel with diesel-like properties. Previously, 1-octanol had been synthesised by (i) the reversal of beta-oxidation (Dellomonaco et al., 2011), (ii) rerouting branched-chain amino acid biosynthesis (Marcheschi et al., 2012) and (iii) extending the 1-butanol pathway (Machado et al., 2012). In this study, we engineer a novel metabolic route for the production of 1-octanol in *Escherichia coli* and furthermore show that it can be naturally excreted into the media.

## 2 Materials and methods

### 2.1 Strains and plasmids

*E. coli* BL21(DE3) was purchased from Novagen. The *E. coli* BL21(DE3)Δ*acrA* strain was constructed by P1 phage transduction, described previously (Datsenko and Wanner), using the BW25113Δ*acrA* strain (The Coli Genetic Stock Center, Yale, USA) as the donor strain. These two strains were engineered using genes encoding for *Mycobacterium marinum* carboxylic acid reductase (CAR), Sfp from *Bacillus subtilis*, *tes3* (thioesterase from *Anaerococcus tetradius* with accession no. EEI82564) and Ahr (an aldehyde reductase previously known as *yjgB*) from *E. coli*. The construct cloning approach was described previously (Akhtar et al., 2013, Kallio et al., 2014). The plasmids pET-TPC3 (encoding Tes3, Sfp and CAR (TPC3, Akhtar et al., 2013, Kallio et al., 2014), assembled as a synthetic operon (Akhtar and Jones, 2009), and pCDF-Ahr_his_ (encoding Ahr with an N-terminal 6xHis-tag) were transformed into chemically competent *E. coli* BL21 (DE3) to generate the TPC3 Ahr strain.

### 2.2 *In vivo* production of fatty alcohols

Strains were cultivated based on a slight modification of the method described previously (Akhtar et al., 2013). LB, containing a 2% (v/v) overnight, LB-based inoculum (30 °C/150 rpm/~18 h), was incubated at 30 °C/150 rpm until it had reached an OD600 of 0.5–0.7. Cells were harvested (17, 000*g*/1 min), washed twice with modified minimal medium (Akhtar et al., 2013) prior to resuspension in the same medium. After addition of isopropyl β-d-1-thiogalactopyranoside (IPTG) at a final concentration of 20 μM, cells were incubated at 30 °C for 14 h. The cellular density was determined from absorbance measurements (attributed to light scattering) at 600 nm (OD_600 nm_) and glucose levels were quantified at 340 nm, based on the reduction of NAD^+^ by glucose-6-phosphate dehydrogenase. Yields were determined by expressing the total production of fatty alcohol (described in detail below) per gram of consumed glucose.

### 2.3 Fatty alcohol quantification

For total fatty alcohol quantification, a 100 μl cell culture was mixed with 200 μl acetone (containing 0.2 mM 1-heptanol as the internal standard). For cellular and extracellular quantification of fatty alcohols, cell cultures were first separated into cellular and media fractions by centrifugation. The cellular fraction was resuspended in 100 μl of water prior to mixing with 200 μl acetone, while the media fraction was directly mixed with 200 μl acetone. All samples were centrifuged and the supernatant transferred to GC vials. Metabolite analysis was performed with an Agilent 7890A gas chromatograph equipped with a 5975 mass spectrometry detector, as described previously (Akhtar et al., 2013). For 1-octanol and 1-hexanol identification, fragmentation patterns and retention times of the analytes were compared with the NIST mass spectral library and commercially available fatty alcohol standards. A standard curve for quantification was prepared with commercially sourced fatty alcohols. Data was normalised with respect to the internal standard and optical density at 600 nm. All given values are an average mean of measurements obtained from at least 6 independent cultures with error bars representing standard error.

## 3 Results

### 3.1 A comparison of the fuel characteristics between fossil-derived diesel and saturated alcohols

Having recently demonstrated the *in vitro* production of a broad range of fatty alcohols based on the activities of CAR and AHR enzymes (Akhtar et al., 2013), we surveyed the literature and compared the fuel characteristics of fossil-derived diesel and saturated fatty alcohols ranging in chain length from 2 to 12 carbons. Several fuel parameters relevant to diesel engines were considered, such as cetane number, viscosity and lubricity (Table 1). Short-chain alcohols such as ethanol and butanol were found to have poor lubricity and high combustibility which would increase engine wear and stress. On the other hand, longer alcohols (>10 carbon atoms long) exhibited high freezing points and extremely low vapour pressures which would render them ineffective in cold weather conditions. In between these two extremes, 1-octanol displayed an energy content, lubricity and viscosity similar to that of petroleum-derived diesel. We concluded that 1-octanol could potentially serve as a suitable fuel target.

**Table 1:** Fuel and physicochemical characteristics of petroleum-derived fuels and its potential substitutes.

	**Energy content (MJ/L)**	**Solubility**^a^**(g/L)**	**Cetane number**^b^	**Lubricity**^c^**(μm corrected wear scar)**	**Viscosity**^d^**cST**	**Density**^a^	**Auto ignition temperature**^b^**(°C)**	**Boiling point**^a^**(°C)**	**Flash point**^a^**(°C)**	**Vapour pressure**^a^**(mmHg)**	**Freezing point**^a^**(°C)**
Methanol	16	Miscible	2	1100	0.6@40 °C	0.79	463	65	11	127	−98
Ethanol	19.6	Miscible	11	603	1.1@40 °C	0.79	420	78	17	55	−114
1-Butanol	29.2	77	17	623	1.7@40 °C	0.81	343	117	29	7	−90
1-Hexanol	31.7	7.9	23	534	2.9@40 °C	0.81	285	158	59	1	−45
1-Octanol	33.7	0.59	39	404	4.4@40 °C	0.83	270	195	81	0.08	−16
1-Decanol	34.6	~0.04	50	406	6.5@40 °C	0.83	255	233	108	<0.1	6
1-Dodecanol	35.3	~0.004	64	345	9.0@40 °C	0.83	275	261	119	<0.1	24
Hydrogenated bisabolene^e^	~37	Immiscible	42	Unknown	2.91	0.82	Unknown	267	111	<0.01	<−78
Biodiesel	32.1	Immiscible	60	314	4–6@40 °C	0.87 (avg)	177–330	315–350	100–170	<1	−3 to −5
Petrodiesel^f^	40.3	Immiscible	45–50	315	1.8–5.8@40 °C	0.84 (avg)	210	150–350	52–96	0.4	−12
Petroleum^g^	32.1	Immiscible	13–17	711–1064	0.4–0.8@20 °C	0.82 (avg)	246–280	27–225	−40	275–475	−60

a Linstrom and Mallard, 2014.

b Harnisch et al. (2013).

c Weinebeck and Murrenhoff (2013).

d Viswanath et al. (2007).

e Peralta-Yahya et al. (2011).

f NREL (2009).

g Louis and Arkoudeas (2012).

### 3.2 Synthesis of 1-octanol

**Fig. 1:**
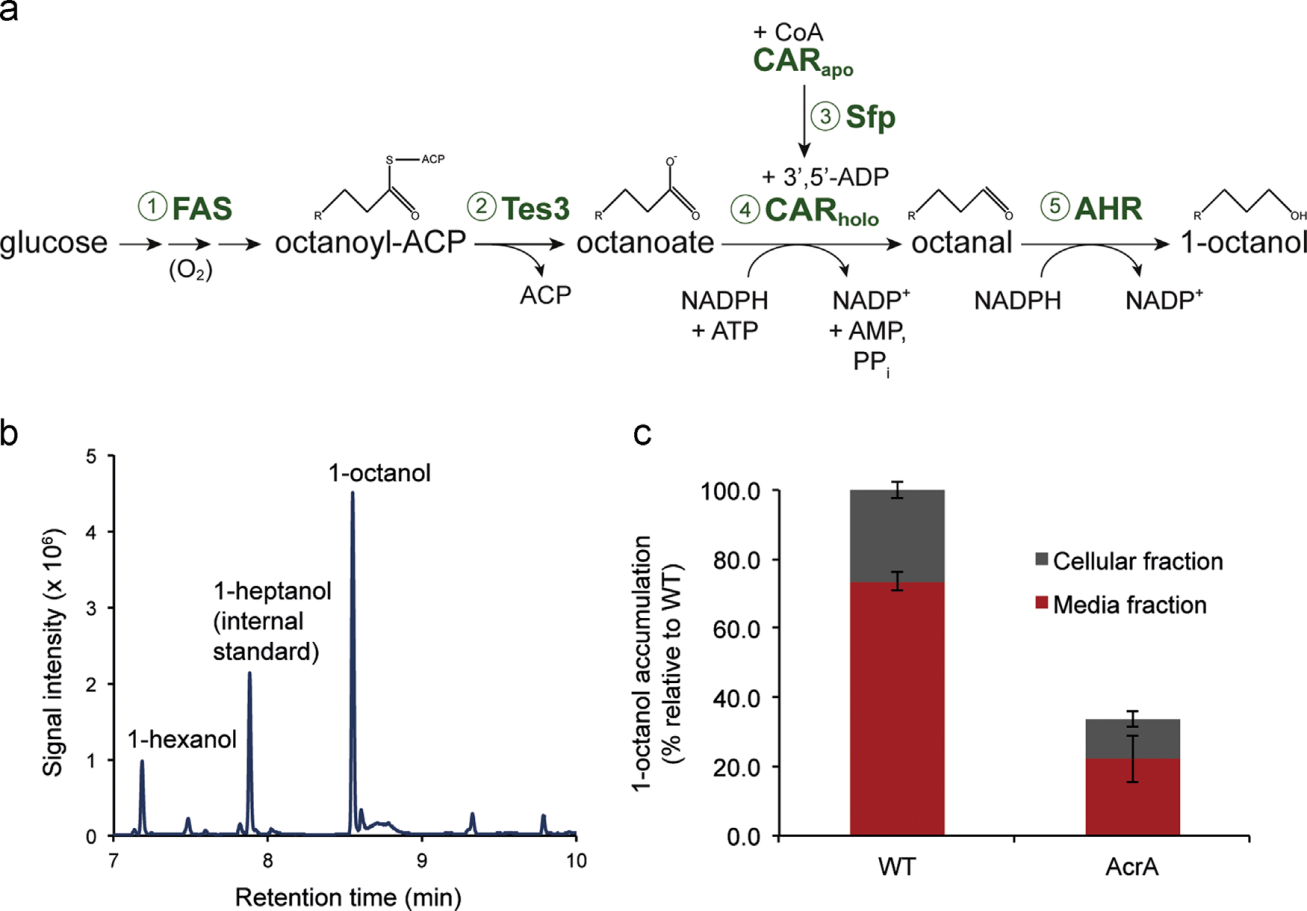
Pathway engineering for the synthesis of 1-octanol. (a) Metabolic scheme depicting the pathway for the production of 1-octanol from glucose and fatty acids in *E. coli*: (1) fatty acid biosynthesis; (2) thioesterase releases free octanoate from FAS; (3) the CAR maturase, Sfp, prepares CAR_holo_ for catalysis; (4) CAR converts the free fatty acid to the corresponding aldehyde and (5) AHR reduces the aldehyde to the corresponding alcohol. (b) Example of GC–MS chromatogram showing the synthesis of 1-octanol. TPC3-Ahr strain was induced in modified minimal medium for 14 h, and analysed for fatty alcohols, as described previously (Akhtar et al., 2013). (c) Distribution of 1-octanol in the two strains *E. coli* BL21(DE3)=WT, and *E. coli* BL21(DE3) Δ*acrA*=AcrA. Abbreviations: Glc, glucose; FAS, fatty acid synthesis; TES, thioesterase; reductase; CAR, carboxylic acid reductase; AHR, aldehyde reductase.

Given its fuel characteristics, we therefore set out to engineer a pathway for the production of 1-octanol. The previously engineered pathway, which resulted in the synthesis of longer chain fatty alcohols (C12-C16), consisted of *E. coli* acyl-ACP thioesterase TesA, *M. marinum* carboxylic acid reductase (CAR), *B. subtilis* phosphopantetheinyl transferase, (Sfp) and *E. coli* Ahr (Akhtar et al., 2013). Since the chain length of the fatty alcohol was determined by the substrate specificity of TesA, this enzyme was therefore replaced with one that had previously been shown to generate the 8-carbon fatty acid precursor, octanoic acid (Jing et al., 2011) (Fig. 1a). Induction of the pathway in the presence of IPTG for 14 hours led to the synthesis of 29(±4) 1-hexanol mg L^−1^ and 62(±3) 1-octanol mg L^−1^ with an average yield of 12(±0.4) mg of 1-octanol g^−1^ glucose and a productivity of 4.4 mg (±0.04) mg 1-octanol L^−1 ^h^−1^ (Fig. 1b; Table 2). No other new metabolites were observed by gas chromatography–mass spectrometry (GC–MS) analysis.

**Table 2:** Comparison of engineered platforms for 1-octanol production in *E. coli*.

**1-Octanol producing system (strain name, mutations, genes induced)**	**Titres of 1-octanol (mg L**^**−1**^**)**	**Yield of 1-octanol**^a^**(mg g**^**−1**^**glucose)**	**Productivity (mg h**^**−1**^** L**^**−1**^**)**	**Cultivation conditions**	**Reference**
BL21(DE3)	62	12	4.4	14 h, 30 °C, shake-flasks	This work
*tes*, *sfp*, *car*, *ahr*				M9-based medium with vitamins and metals^b^	
MG1655	100	25	2.1	48 h, 37 °C, shake-flasks	Dellomonaco et al. (2011)
*fadR ato*C(con) ∆*arcA* ∆*crp*::*crp*^c^ ∆*adhE* ∆*pta*				M9-based medium with vitamins and metals^b^	
∆*frdA* ∆*fucO* ∆*yqhD* ∆*fadD fadBA betA*					

BW25113	70	Not applicable	1.5	48 h, 30 °C, culture tubes	Machado et al. (2012)
F^0^ [*traD36*, *proAB*+, *lacI*^*q*^*Z*Δ*M15* (*tetR*)]					
Δ*ldhA* Δ*adhE* Δ*frdBC* Δ*pta*				Terrific broth^b^	

ATCC98082 (threonine overproducing strain)	15	Not given	0.04	48 h, 30 °C, shake-flasks	Marcheschi et al. (2012)
Δ*rhtA thrA*, *thrB*, *thrC*, *ilvA*, *leuA*,					
*leuA*^b^*leuB*, *leuC*, *leuD*, *kivD*, *adh6*				M9 medium with yeast extract^b^	

a Maximum possible theoretical yield is 361 mg 1-octanol per gram of glucose.

b Supplemented with glucose.

c Modified gene.

### 3.3 Excretion of 1-octanol

We noted that during the early to late stationary phases of cell cultivation with TPC3-Ahr, a strong perfume-like fragrance emanated from the cell cultures which suggested the extracellular presence of product(s). After separation of the cells from the media, GC–MS analysis confirmed that 73±3% of the total 1-octanol in the culture was present in the media while the remainder was found to be localised within the cellular fraction (Fig. 1c). *E. coli* harbours a large range of efflux pumps (Segura et al., 2012). Among them, the AcrAB–TolC complex is the most well-studied and has been implicated in tolerance to exogenously added solvents (Tsukagoshi and Aono, 2000, White et al., 1997). We constructed an Δ*acrA* deletion strain of *E. coli* BL21(DE3) in order to test whether AcrAB–TolC was responsible for the efflux of internally produced 1-octanol. The mutant strain displayed a ~3-fold decrease in excreted 1-octanol along with a similar drop in total 1-octanol production levels (Fig. 1c). The ratio of external to internal 1-octanol, however, was slightly lower for the Δ*acrA* deletion strain (2.0) compared to the wild-type strain (2.8). The marked reduction in both excretion and production suggested that the AcrAB–TolC complex is most likely responsible for the majority of 1-octanol efflux.

## 4 Discussion

On account of its diesel-like characteristics, 1-octanol is an attractive biofuel target. In comparison to petrodiesel, its lower vapour pressure could reduce transportation and storage hazards, while its higher auto-ignition temperature could raise the air:fuel compression ratios and in turn improve fuel combustion efficiency. Real simulation tests with compression ignition engines have shown that 1-octanol can reduce particulate matter by as much as 20-fold in comparison to petrodiesel (Heuser et al., 2013). In the case of alternative fuels such as biodiesel, inefficient combustion can lead to the formation of hygroscopic by-products such as fatty acids, mono- and di-glycerides which can promote engine corrosion (Atabani et al., 2012). Furthermore, the cold flow properties of biodiesel are inferior to 1-octanol, (*e.g.* pour point; −9 °C *vs* −13.5 °C) making the latter a potentially better fuel to handle and operate under cold conditions, at least as a blending component.

In this study, we engineered a pathway that utilised precursors from the natural process of fatty acid synthesis. The octanoic acid precursor was generated by incorporating a fatty acyl-ACP thioesterase which was previously shown to have a preference for fatty acyl-ACP chains of 6–8 carbon atoms. Although the yield (62 mg L^−1^) was found to be ~2-fold lower than the Dellomonaco et al. (2011) study, the CAR-based platform required a considerably shorter cultivation period (14 h) for optimal production of 1-octanol (Table 2). Thus, the productivity (4.4 mg1-octanol L^−1 ^h^−1^) exceeded previously obtained values of 2.1, 1.5 and 0.04 mg 1-octanol L^−1 ^h^−1^ (Dellomonaco et al., 2011, Machado et al., 2012, Marcheschi et al., 2012; Table 2) without resorting to host engineering or advanced bioprocessing techniques. From a stoichiometric viewpoint, since 2 moles of glucose (MW=180) would be required for the synthesis of 1 mole of 1-octanol (MW=130), the maximum theoretical yield possible is 361 mg 1-octanol per gram glucose. Clearly then, the low yields obtained in this and previous studies indicate that there is still considerable room for metabolic improvements. We expect that optimisation of the process *via* fatty acid engineering (Torella et al., 2013), modulated levels of efflux pumps (Wang et al., 2013), regulated metabolic flux (Dahl et al., 2013) and *in situ* product removal techniques (Baez and Shiloach, 2013) is likely to improve 1-octanol productivity, yields and titres even further.

A major advantage of biologically producing 1-octanol as an end-product lies in its propensity for extracellular localisation. This trait is advantageous for downstream purification of the end-product. In contrast, fuels such as biodiesel become confined to the internal cellular compartments due to their rather large structure and intracellular role as a carbon storage medium (Kalscheuer et al., 2006). Energy-intensive extraction methods are therefore typically required to harvest, extract and isolate biodiesel from the host organism (Chisti, 2013). As suggested in this work, the extracellular localisation of 1-octanol is most likely facilitated by the AcrAB–TolC complex. The importance of TolC, though not AcrAB, for the internal production of free fatty acids (C8–C14) was recently observed in a study in which excretion unfortunately was not monitored (Lennen et al., 2013). Similarly, the over-expression of native *E. coli* transporters or transporter components have previously been found to stimulate either excretion (Doshi et al., 2013) or both excretion and production (Wang et al., 2013) of native or non-native products. These studies on the role of TolC-dependent transporter complexes suggest that the elucidation of cause-and-effect may be complicated by their (1) overlapping substrate specificities and (2) complex sub-unit stoichiometry; such properties are likely to alter the dynamics of efflux in very subtle ways and make difficult the task of determining the efflux contribution of any single transporter complex. In addition, though not yet studied, it is possible that the overlapping susbstrate specificities may well serve complementary roles depending upon the physiological state of the cell.

Despite the complexity of these experimental systems, the simultaneous reduction in both excretion and total production of 1-octanol in the Δ*acrA* mutant, as well as previous work (Lennen et al., 2013, Wang et al., 2013), suggest that efflux pumps can have a substantial impact on the total flux of metabolic pathways that lead to a product with no obvious storage role within the cell. Studies are currently underway to shed further light on the correlation between excretion and production, as well as elucidating the entire complement of efflux pumps responsible for the excretion of fatty fuels of varying chain-lengths.

In summary, given the biological feasibility of its production, along with its natural excretion within the media and diesel-like fuel characteristics, we propose 1-octanol to be an attractive metabolic target for fuel-related applications.
